# Supplementary material for: High coverage COVID-19 mRNA vaccination rapidly controls SARS-CoV-2 transmission in long-term care facilities
Source: Commun Med (Lond). 2021 Jul 16;1:16. doi: 10.1038/s43856-021-00015-1 (PMC9053242; doi:10.1038/s43856-021-00015-1)
Supplement: Supplementary file 1 — Supplementary material. [file 43856_2021_15_MOESM1_ESM.pdf]

Supplementary Information for: *"High coverage COVID-19 mRNA vaccination rapidly controls SARS-CoV-2 transmission in Long-Term Care Facilities"*

Pablo M De Salazar[1,#]*, Nicholas Link,* [2-5,#]*, Karuna Lamarca*[6]*, Mauricio Santillana*[1,3-5]

1. Center for Communicable Disease Dynamics, Department of Epidemiology, Harvard TH Chan School of Public Health, Boston, United States
2. Department of Biostatistics, Harvard TH Chan School of Public Health, Boston, United States
3. Machine Intelligence Lab, Boston Children's Hospital, Boston, United States
4. Computational Health Informatics Program, Boston Children's Hospital, Boston, United States
5. Department of Pediatrics, Harvard Medical School, Harvard University, Boston, United States
6. Home Hospitalization Unit, Department of Infectious Diseases, Dos de Maig Hospital, Universitat Autònoma de Barcelona, Barcelona, Spain

# These authors contributed equally

Supplementary Methods

Details on ascertainment standards:
Starting in July 2020, LTCFs in Catalonia implemented rigorous COVID-19 surveillance that did not solely rely on detection of symptomatic cases[1] .All contacts among staff and residents are screened using molecular test (PCR or antigen test) immediately upon confirmation of an index infection in a facility; further, all staff and residents are regularly screened independently of whether the individuals show symptoms or not; public health guidelines require to screen staff every 2-4 weeks depending on the population size where the LTCFs is located. Thus, the amount of documented infection relies on all infections but over symptomatic infections (which have been estimated to be around ~60% in LTCFs/skilled nursing homes)[2-3]. For our analysis using this dataset, we assume that the number of documented infections and COVID-19 deaths closely approximates the true number of infections and deaths. For example, in a facility where individuals are screened every 2 weeks[4] on top of having symptomatic surveillance and outbreak investigation standards, reporting no documented infections --when in reality there is transmission-- would require the full outbreak to remain undetected (i.e., all infections occurring within the same outbreak remain asymptomatic), the outbreak to die off within days or weeks without isolation of exposed individuals, and the infections to show no detectable viral RNA in <14 days after infection (the median duration of detectable SARS-CoV-2 in unvaccinated individuals is estimated to be ~20 days[5]). Similarly, we assume that the event of not detecting any infection in any of the facilities of a county for a whole week closely approximates the true absence of transmission in the county. Deviations from these assumptions are discussed in the main text.

Summarized description and justification of inference models:

We use three different models to study three different processes/outcomes: (1) the number of infections/cases in long term care homes, (2) the number of deaths in long term care homes, and (3) the probability of any infection occurring within any long-term care home in a county. For (1), to model the number of infections in LTCFs we use a negative binomial regression, which is standard for modeling case count data. For (2), to model the number of deaths in LTCFs, we use a zero-intercept linear regression, since the relationship of deaths and cases appears to be monotonic and linear -and we assume no covid-19 attributable death would happen in the absence of infections. Model (3) is where we use the logistic regression model to estimate

binary "transmission events" at the county level. We defined a transmission event if there was any reported infection in that county for that week (i.e., = 1 if there was at least one new infection and = 0 if there were no new infections). Since the outcome is binary and we wish to generate predictions of the probability of this outcome occurring, a logistic regression approach was chosen. We analyzed this outcome since it is indicative of the efficacy of vaccines reducing SARS-CoV-2 transmission. This is because without a reduction in transmission, we would expect to see a reduction in the total number of infections proportional to the number of people vaccinated, but we wouldn't expect to see a 100% reduction in infections. Using the binary outcome and the logistic regression model allows us to analyze if there are more county-level 100% reductions in infections than we would expect.

Estimating infections and deaths averted by vaccinations

To estimate the total infections and deaths averted, we aggregate data across all of Catalonia. While it might make sense to use point estimates of infections and deaths averted from individual counties, the more granular model fits are not as good and there is not a clear way to combine the uncertainty from these multiple estimates given their temporal and spatial correlation. We trained a negative binomial model (model 1, eq. 1) to predict the nursing home infections, N_d, for day *d* using community infections that day and one week prior, C_d and C_{d-7} respectively. N_d and C_d are moving weekly averages of documented infections.

$log(E[N_d]) = B_0 + B_1 log(1 + C_d) + B_2 log(1 + C_{d-7}) + log(S_d)$ (eq. 1)

where $S_d = 1 - Im_d$ andIm_d is the proportion of the population that is immune:

$$Im_0 = 0.1$$
$$Im_d = max(0, Im_{d-1}(1 - 0.18/365) - D_{d-1}/n + N_d/n)$$

We include an offset term $log(S_d)$, where S_d is the proportion of the population that is susceptible, to account for the expected decrease in infections due to susceptible depletion; including $log(S_d)$ amounts to multiplying predictions by the susceptible proportion. $D_d$is the weekly average of deaths on day *d*, the $(1 - 0.18/365)$ term accounts for a 18% turnover rate in nursing homes in a usual year [6], and n is the estimated nursing home population (n=57,922). Since the exact population size in LTCFs was not available at all considered spatial resolutions, we approximated this quantity by the maximum number of vaccinated people once vaccination was reported complete (n=57,922).

For deaths, we use a similar model (model 2, eq. 2), though we use a zero-intercept linear regression of community infections because the relationship between number of infections and number of deaths is likely linear (because a specific proportion of nursing home infections weeks prior will be expected to die).

$E[D_d] = B_1 C_d + B_2 C_{(d-7)} + B_3 C_{(d-14)} + B_4 C_{(d-21)}$ (eq. 2)

The model was trained on data from a baseline period, July 6, 2020 to December 27, 2020, and then applied to data from an evaluation period, December 28 to March 28 (on December 26, nursing homes began administering vaccines). We generated model fits and prediction intervals in the evaluation period. The prediction intervals for deaths (eq. 2) were generated by using R's prediction function and the prediction intervals for infections (eq. 1) were generated using a parametric bootstrap procedure. To estimate the number of infections or deaths averted during a target period, we summed the daily model fits and bounds of the prediction intervals. This yields wider intervals than is likely true but given the temporal correlation of the predictions it

would not be sensible to combine the variances of each prediction as if they were independent. We selected two target periods to compute averted infections and deaths based on the dates when 70% of the nursing homes residents received the first vaccine dose and when the same proportion received the second dose January 14 and February 6. This allowed us to adjust for changes over time of the vaccines' effect given the delay between vaccination and efficacious immunization, as well as to account for vaccine coverage close to herd immunity estimates (assumed >70%, see main text). As a supplementary analysis (see Supplementary Analysis bellow), we estimated the number of deaths per LTCFs infection, which approximates the mortality rate, before and after vaccination.

Predicting change in probability of detected transmission in facilities

We analyzed the changes in detected transmission at the county level. We define the binary outcome of detected transmission occurrence, O_{iw}, as at least one COVID-19 infection among nursing homes residents in county *i* for week w. We predict the probability of a transmission occurrence, $\pi_{iw} = P(O_{iw})$, using the logistic regression model (model 3, eq.3).

$$logit(\pi_{iw}) = B_0 + B_1 C_{iw} + B_2 C_{i(w-1)} + log(S_{iw}) \quad \text{(eq. 3)}$$

Counties without any detected transmission during the pre-vaccination period (1 county) or without any weeks with no transmission (4 counties) were excluded from this analysis because of the inability to fit a logistic regression model with only one outcome class. To avoid overfitting, we calculated leave-one-out predictions during the pre-vaccination period and out-of-sample predictions for the vaccination period using a model trained in the pre-vaccination period. Then we computed the ratio of observed to predicted transmission events (denoted as $TD_w = Transmission - Deviation\ (week\ w)$) as an approximation of the vaccine effectiveness in fully preventing transmission.

$$TD_w = \frac{\sum_i O_{iw}}{\sum_i \pi_{iw}} \quad \text{(eq. 4)}$$

To generate confidence intervals around our predictions, we calculated the sample standard deviation (σ_{TD}) of the pre-vaccination predictions (around a mean ≈1) and use the normal distribution confidence intervals. We believe this is reasonable because we are summing 36 (41 - 5 excluded counties) individual (non-normal) distributions, and by the central limit theorem this should be approximately normal. To estimate the proportion of documented transmission averted with 90% confidence intervals, we calculated

$$1 - TD_w(1 - 1.645\sigma_{TD} - TD_w, 1 + 1.645\sigma_{TD} - TD_w).$$

Estimating changes in the fatality rates

As sensitivity, we predicted the number of LTCFs deaths from LTCFs documented infections using the model 4 (eq. 5) in Catalonia.

$$D_{rd} = B_1 N_{r(d-7)} + B_2 N_{r(d-14)} + B_3 N_{r(d-21)} + \epsilon_d \quad \text{(eq. 5)}$$

This is a proxy for the fatality rate per infection. Interestingly, the fatality rates seem to increase in January (Supplementary Figure 1) after vaccines are first delivered and decrease later on, which could explain the excess of deaths observed in the main analysis compared to those predicted from community infections.

Percent errors for documented infections and deaths models

As sensitivity we computed the percent error over time for documented infections (model 1, eq. 1) and deaths (model 2, eq 2). As shown in Supplementary Figure 2 the trend consistently increased toward negative values after vaccination started. For both events, the deviation predictions vs observations during the target time period Feb 6- March 28 becomes close to 100%.

All analysis was conducted using R 4.0.3 (https://www.R-project.org/).

	Since 70% first-dose vaccination - Jan 14, 2021 (90% CI)	Since 70% second-dose vaccination - Feb 6, 2021 (90% CI)
All of Catalonia<br>*% averted (90% CI)*	1659 (0, 4817)<br>*42% (0%, 68%)*	1371 (157, 2867)<br>*75% (36%, 86%)*
Alt Pirineu i Aran	109 (0, 469)	72 (0, 283)
Barcelona Ciutat	174 (0, 753)	191 (0, 491)
Camp de Tarragona	49 (0, 685)	87 (0, 304)
Catalunya Central	255 (0, 836)	195 (0, 527)
Girona	190 (0, 843)	117 (0, 429)
Lleida	167 (0, 521)	134 (0, 354)
Metropolitana Nord	256 (0, 854)	311 (61, 644)
Metropolitana Sud	280 (0, 798)	182 (0, 437)
Terres de l’Ebre	50 (0, 243)	11 (0, 69)

Supplementary Table 1. Number of documented infections averted. Showing the number of predicted averted cases in all of Catalonia and in each healthcare area. All values are cumulative estimates of cases averted between the starting dates, Jan 14, 2021 and Feb 6, 2021, and March 28, 2021. The 90% confidence intervals are the sums of the bounds of the daily 90% prediction intervals, so they are likely wider than in reality. Negative confidence interval values (indicating higher-than-expected cases) are truncated at 0.

	Since 70% first-dose vaccination - Jan 14, 2021 (90% CI)	Since 70% second-dose vaccination - Feb 6, 2021 (90% CI)
All of Catalonia<br>*% averted (90% CI)*	382 (55, 709)<br>*38% (8%, 53%)*	445 (220, 669)<br>*74% (58%, 81%)*
Alt Pirineu i Aran	117 (49, 185)	64 (17, 110)
Barcelona Ciutat	56 (0, 135)	67 (13, 121)
Camp de Tarragona	12 (0, 77)	31 (-14, 75)
Catalunya Central	37 (0, 89)	56 (19, 92)
Girona	44 (0, 112)	58 (11, 105)
Lleida	20 (0, 51)	18 (-4, 39)
Metropolitana Nord	60 (0, 128)	71 (24, 119)
Metropolitana Sud	54 (0, 121)	74 (28, 120)
Terres de l’Ebre	30 (10, 50)	15 (1, 29)

Supplementary Table 2. Number of deaths averted. Showing the number of predicted averted deaths in all of Catalonia and in each healthcare area. All values are cumulative estimates of deaths averted between the starting dates, Jan 14, 2021 and Feb 6, 2021, and March 28, 2021. The 90% confidence intervals are the sums of the bounds of the daily 90% prediction intervals, so they are likely wider than in reality. Negative confidence interval values (indicating higher-than-expected cases) are truncated at 0.

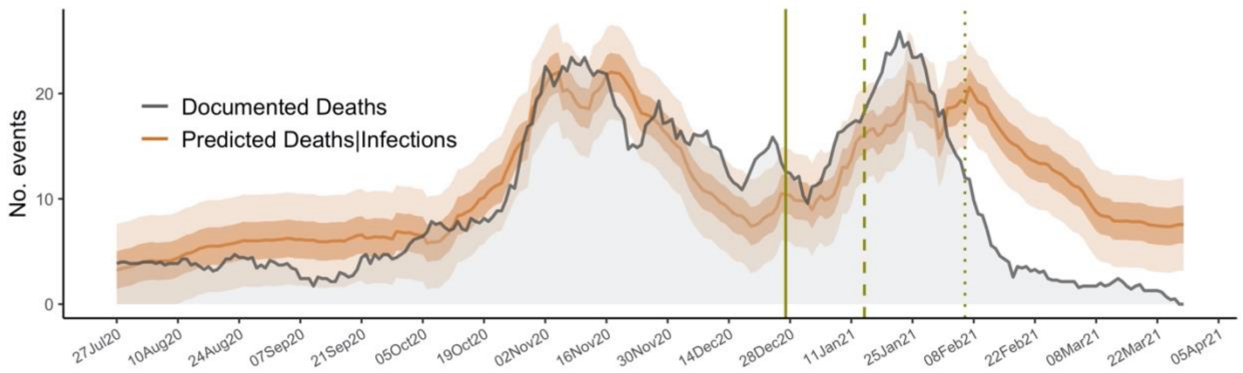

Supplementary Figure 1. Predictions of the number of LTCFs deaths from LTCFs documented infections using the model 4 (eq. 5). The grey lines are the documented deaths, and the brown lines are the predicted deaths, with ribbons for the 50%PI and 90% PI. Vertical lines show key analysis time points: when vaccination started (solid), when 70% of residents received the first dose and when 70% of residents received the second dose.

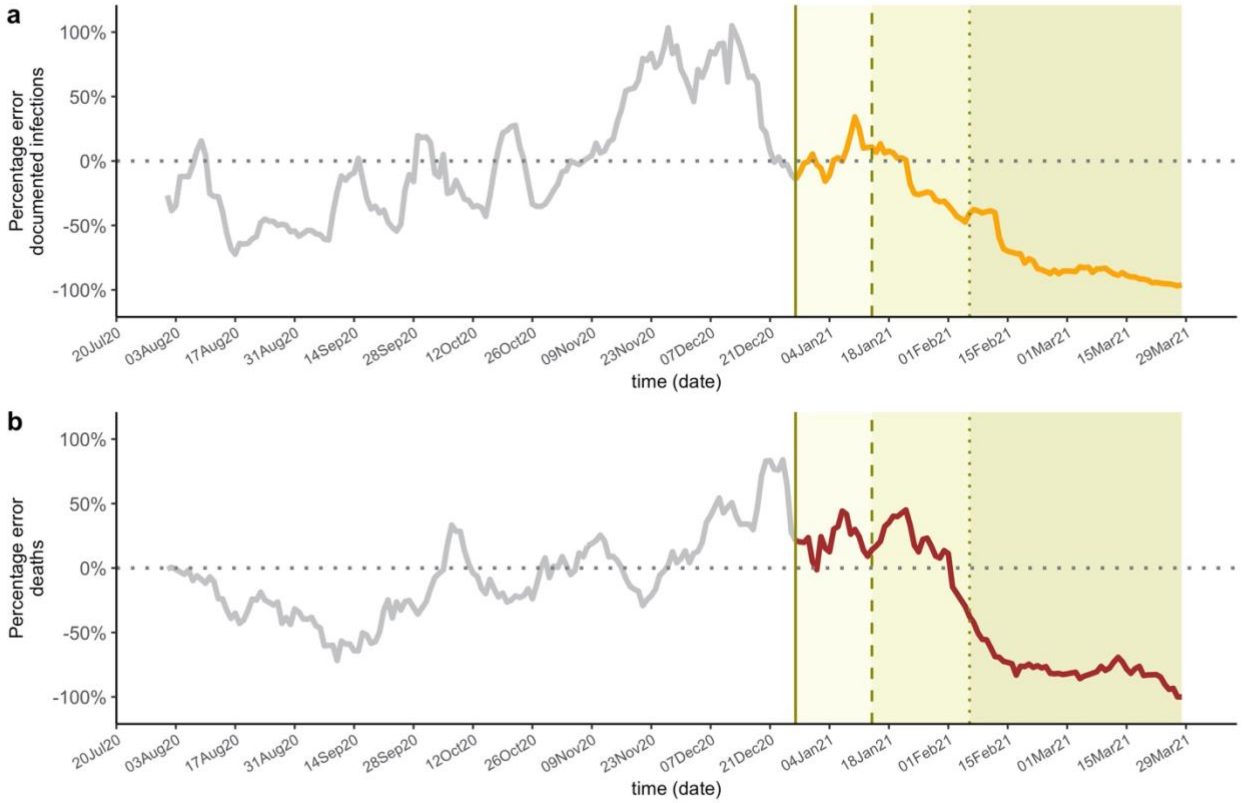

Supplementary Figure 2. Percent errors for documented infections and deaths models**.** Solid lines represent daily percentage error for documented infections (a) and death model (b). Grey color is used for the training time period pre vaccination and orange and red once vaccination started. Vertical lines show key analysis time points: when vaccination started (solid), when 70% of residents received the first dose and when 70% of residents received the second dose

Supplementary References

1. Lipsitch, M. & Dean, N. E. Understanding COVID-19 vaccine efficacy. *Science* **370**, 763–765 (2020).
2. Polack, F. P. *et al.* Safety and Efficacy of the BNT162b2 mRNA Covid-19 Vaccine. *N. Engl. J. Med.* **383**, 2603–2615 (2020).
3. Baden, L. R. *et al.* Efficacy and Safety of the mRNA-1273 SARS-CoV-2 Vaccine. *N. Engl. J. Med.* **384**, 403–416 (2021).
4. Moustsen-Helms, I. R. *et al.* Vaccine effectiveness after 1st and 2nd dose of the BNT162b2 mRNA Covid-19 Vaccine in long-term care facility residents and healthcare workers – a Danish cohort study. doi:10.1101/2021.03.08.21252200.
5. Hall, V. J. *et al.* Effectiveness of BNT162b2 mRNA Vaccine Against Infection and COVID-19 Vaccine Coverage in Healthcare Workers in England, Multicentre Prospective Cohort Study (the SIREN Study). *SSRN Electronic Journal* doi:10.2139/ssrn.3790399.
